# Comparison of preferences of healthcare professionals and MS patients for attributes of disease‐modifying drugs: A best‐worst scaling

**DOI:** 10.1111/hex.12599

**Published:** 2017-07-21

**Authors:** Ingrid E. H. Kremer, Silvia M. A. A. Evers, Peter J. Jongen, Mickaël Hiligsmann

**Affiliations:** ^1^ Department of Health Services Research Care and Public Health Research Institute (CAPHRI) Maastricht University Maastricht The Netherlands; ^2^ Center for Economic Evaluations Trimbos Institute Netherlands Institute of Mental Health and Addiction Utrecht The Netherlands; ^3^ MS4 Research Institute Nijmegen The Netherlands; ^4^ Department of Community & Occupational Medicine University Medical Centre Groningen Groningen The Netherlands

**Keywords:** multiple sclerosis, best‐worst scaling, healthcare professionals, disease‐modifying drugs, shared decision making, patients

## Abstract

**Background:**

The choice between disease‐modifying drugs (DMDs) for the treatment of multiple sclerosis (MS) becomes more often a shared decision between the patient and the neurologist and MS nurse. This study aimed to assess which DMD attributes are most important for the healthcare professionals in selecting a DMD for a patient. Subsequently, within this perspective, the neurologists’ and nurses’ perspectives were compared. Lastly, the healthcare professionals’ perspective was compared with the patients’ perspective to detect any differences that may need attention in the communication about DMDs.

**Design:**

A best‐worst scaling (BWS) was conducted among 27 neurologists and 33 MS nurses treating patients with MS to determine the importance of 27 DMD attributes. These attributes were identified through three focus groups with MS patients in a previous study (N=19). Relative importance scores (RISs) were estimated for each attribute. Multivariable linear regression analyses were used to compare the different perspectives.

**Results:**

According to the neurologists and nurses, safety of the DMD was the most important DMD attribute in the treatment decision, closely followed by effect on disability progression, quality of life and relapse rate. Patients with MS agreed with the importance of the last three attributes, but valued safety significantly lower (*b*=−2.59, *P*<.001).

**Conclusions:**

This study suggests that, overall, neurologists and nurses regard the same DMD attributes as important as MS patients with the notable exception of safety. This study provides valuable information for the development of interventions to support shared decision making and highlights which attributes of DMDs may need additional attention.

**Key points**

- Understanding the healthcare professionals’ perspective about the importance of attributes of disease modifying drugs for multiple sclerosis, and comparing this perspective with the patients’ perspective, would be important to develop a patient decision aid that is accepted by, and usable for, both patients and healthcare professionals in the shared decision‐making process
- The healthcare professionals and patients overall agree about the importance of the effects of DMDs on disability progression, quality of life and relapse rate, but safety was valued significantly lower by patients.
- The study results provide guidance for the selection of information to be included in a patient decision aid for disease‐modifying drugs in multiple sclerosis to effectively support the patient and healthcare professional in making a shared decision.

## 1 INTRODUCTION

Multiple sclerosis (MS) is a degenerative disease of the central nervous system causing physical and cognitive disabilities.^1^ The relapsing‐remitting form of MS (RRMS) can be treated with disease‐modifying drugs (DMDs) to reduce progression or even induce improvements. DMD treatment requires long‐term administration with a minimum of missed doses. DMDs are classified as first‐, second‐ or third‐line treatments. First‐line DMDs are used in patients with mild disease activity. In case of high disease activity or intolerance to the side effects, second‐ or third‐line DMDs may be chosen. These DMDs have more favourable effectiveness rates but less favourable safety profiles.^2^, ^3^ Overall, thirteen DMDs are currently approved by the European Medicine Agency and the Food and Drug Administration.^4^, ^5^ This variety in DMDs often provides MS patients with more than one acceptable option for treatment.

Therefore, a choice between DMDs needs to be made, which is often difficult. Besides differences in effectiveness rates and safety profiles, the DMDs can differ in other characteristics, or attributes, such as mode of administration and side effects. To make an optimal choice, the patient's preferences for such attributes should be incorporated in the decision. In shared decision making, the patient makes the decision about the treatment together with the healthcare professional.^6^ The healthcare professional ensures the patient is informed about the best available treatment options and supports the patient in clarifying the advantages and disadvantages of each of the options. The patient communicates his or her values and preferences for these advantages and disadvantages, and deliberates with the healthcare professional, in this case a neurologist and/or an MS nurse, about the treatment options to reach a decision. Decision aids, which are specifically designed to support shared decision making, have been shown to improve the patients’ involvement in the decision and the quality of the decision.^7^

Understanding the preferences of patients and healthcare professionals for treatment options could inform the development of a decision aid. Although the treatment decision should be individualized to every patient and should include the patient's preferences and healthcare professional's opinion tailored to the patient's needs, the evaluation of the average perspectives about the importance of the treatment attributes in the decision could be useful for developing a patient decision aid that meets most patients’ and healthcare professionals’ informational needs. An assessment of the average preferences could be valuable for selecting the information that needs to be included in the decision aid to ensure effectively supporting the shared decision‐making process without making the use of the decision aid too cognitively burdensome for the patient. Preferences of patients for attributes of DMDs have been evaluated in several stated preference studies.^8^, ^9^, ^10^, ^11^, ^12^, ^13^, ^14^, ^15^, ^16^ Recently, focus groups and a best‐worst scaling were conducted to identify and prioritize a range of attributes that could be important in clinical decision making from the patients’ perspective.^8^ This study found that the average MS patient regarded the effects on disability progression and quality of life as the most important attributes of DMDs in the decision, followed by the effect on the relapse rate, severity of side effects and the effect on the severity of relapses.^8^ Several studies showed that preferences of healthcare professionals for treatment options can differ from the preferences of patients.^17^, ^18^, ^19^, ^20^, ^21^ These differences in preferences could result in different expectations and information needed from the decision aid. In MS, one study has assessed the neurologists’ perspective regarding the importance of a limited number of five attributes in the DMD choice.^22^ To our knowledge, the patients’ perspective on important DMD attributes for choosing between DMDs has not yet been directly compared with the healthcare professionals’ perspective.

This study aimed first to use a rigorous method, a best‐worst scaling, to identify which DMD attributes the healthcare professionals (neurologists and MS nurses) find important to take into consideration in the decision regarding DMD treatment. As the relationship with the patient may differ between neurologists and MS nurses because of the more frequent and informal contact between MS nurses and patients, the second aim of this study was to evaluate whether the perspectives on the importance of DMD attributes differ between neurologists and MS nurses. Third, the overall MS healthcare professionals’ (neurologists and MS nurses) perspective was compared with the MS patients’ perspective on the importance of DMD attributes in the treatment decision.

## 2 METHODS

**Figure 1:**
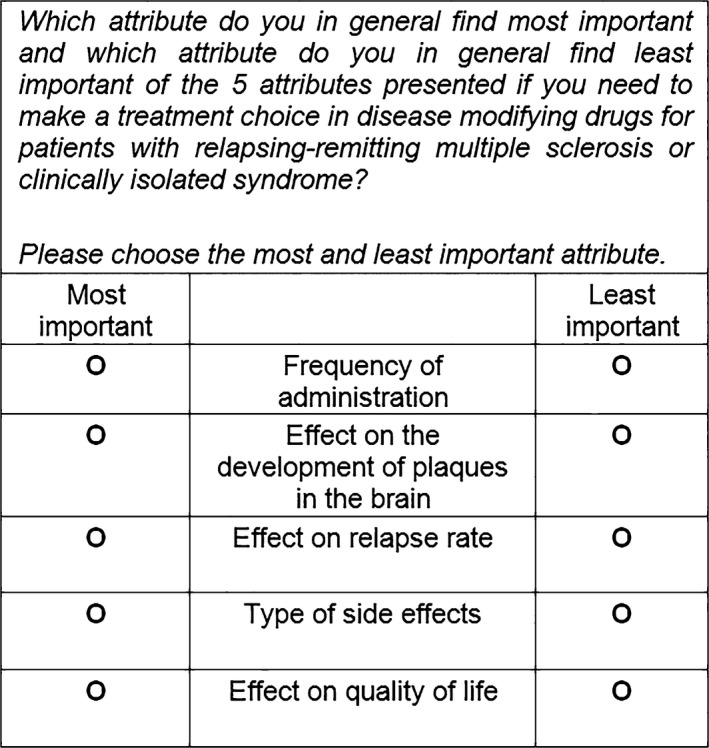
Example of a choice task in the best‐worst scaling

A best‐worst scaling case 1 as described by Flynn and Marley^23^ was conducted to obtain the neurologists’ and MS nurses’ perspective on the importance of attributes of DMDs. In contrast to discrete choice experiments, a best‐worst scaling allows for obtaining the relative importance of attributes for a large number of attributes, regardless of the levels of the attributes.^24^ The best‐worst scaling was embedded in an online questionnaire. Therefore, the Checklist for Reporting Results of Internet E‐Surveys was followed for reporting the design and the results.^25^ We used the same best‐worst scaling that was performed to elicit preferences for patients with MS.^8^ The best‐worst scaling included 27 attributes, which were identified in a two‐stage procedure. First, in an exploratory phase, possible relevant attributes were identified through a literature review and interviews with three neurologists and three nurses. Second, three focus groups (N=19) were conducted to elicit the attributes that patients with MS regarded important in the decision. At the end of each focus group, any additional attributes from the exploratory phase that were not mentioned yet were discussed to evaluate whether these additional attributes were relevant as well. A total of 27 important attributes for the decision about DMD treatment were identified. Using Sawtooth SSI Web version 8.2.0, four best‐worst scaling versions, each consisting of 17 unique choice tasks with five attributes per choice task, were created to obtain the most efficient, fractional design. In such a design, it is not needed to include all possible combinations of attributes in the choice tasks, which is far too burdensome to administer. Instead, specific sets of five attributes from the full attribute lists are created to ensure that the relative importance of each attribute can be derived. Each attribute was presented 12 or 13 times in the four best‐worst scaling version, was combined at least once with every other attribute and appeared two to four times in each position in the choice task. Each respondent randomly received one of the best‐worst scaling versions. In every choice task, the respondent was asked to state which attributes were the most and the least important in the decision about DMDs, making a hypothetical trade‐off between the attributes.^24^ The best‐worst scaling was pilot tested among researchers (n=3) and patients with MS (n=3) to ensure comprehensibility of the questionnaire. An example of a choice task for neurologists and MS nurses is presented in Figure 1. Further description on the identification, selection and definition of attributes and on the development of the choice tasks and the questionnaire is described in detail elsewhere.^8^

In addition to the best‐worst scaling choice tasks, questions about demographical and professional characteristics were included. The respondents had to answer all questions to complete the questionnaire. At the end of the questionnaire, an optional open question provided the respondents the opportunity to report any important attributes in the decision about DMD treatment they missed in the choice tasks of the best‐worst scaling.

### 2.1 Subjects

Healthcare professionals were eligible for participation in the study if they were neurologists (i.e. MS‐specialized neurologists or general neurologists) or MS nurses (i.e. MS‐specialized nurses or nurse practitioners, physician assistants or nurse consultants specialized in MS) and involved in the treatment of patients with MS in the Netherlands. These healthcare professionals generally work in hospitals. The Netherlands counts about 80 (academic or general) hospitals. The neurological department of a hospital generally consists of multiple neurologists of which some neurologists may have one or a few specific areas of focus. The exact number of MS neurologists in the Netherlands is unknown, but most departments have one, two or three neurologists who focus on the treatment of patients with MS. About 135 MS nurses were working in the Netherlands at the time of the study.

Neurologists with a focus on the treatment of MS were identified by searching websites of all hospitals in the Netherlands. We compiled a mailing list for 120 neurologists through the network of one of the authors (PJ)—including the MS working group of the Dutch Neurologists Association and the MSmonitor Working Group—or through personal contact with the hospital. The neurologists were asked to participate through an email explaining the purpose of the study and containing a direct link to the online questionnaire. Additionally, an extra reminder email, in which the neurologists were personally addressed, was sent to 52 members of the MS Working Group of the Dutch association for neurology and the MSmonitor Working Group, of which six neurologists responded to have completed the questionnaire earlier and four neurologists responded to have completed the questionnaire after the email was sent.

Nurses were contacted through a call on the communication system of the professional association for MS nurses to which about 150 MS nurses and other nurses with an interest in MS had access and, additionally, through an email to 88 nurses to increase response. MS nurses for this mailing list were identified via an inventory of MS caregivers and healthcare institutions published on a patient information website about MS endorsed by a Dutch patient association. Additionally, all nurse practitioners and physician assistants in MS (N=15) were also contacted through a mailing list of their professional association. In case of no response among the neurologists and nurses, a reminder email was sent after 1 and 2 weeks after the first email. The neurologists and nurses were informed that by completing the questionnaire they gave consent for their responses to be used in the study. Cookies were used to prevent the same respondent from filling out the questionnaire twice. The study was fully anonymous, and no data, such as IP addresses, were recorded that could lead to the identity of the healthcare professional.

### 2.2 Analyses

Only completed questionnaires were included in the analyses. Descriptive statistics were used to present characteristics of the neurologists and MS nurses. To evaluate the preferences of the healthcare professionals for the DMD attributes, Sawtooth SSI Web version 8.2.0 was used to perform a hierarchical Bayes model employing a multinomial logit procedure.^26^ A raw score for the importance of each attribute was calculated in an iterative process of estimating each respondent's utility score conditionally on estimates of other respondents’ utility scores. The raw score was rescaled to a relative importance score (RIS) on a ratio scale.^27^ The RIS of an attribute represents the importance of the attribute in relation to the importance of the other attributes in the decision about the DMD treatment of MS according to the professionals. The RISs of all 27 attributes for each respondent sum up to 100. Therefore, if there would be no difference in importance of the attributes, each attribute would obtain a RIS of 3.7. Deviations from this score would suggest that some attributes are more important in the treatment decision than others. To ensure only responses of neurologists and MS nurses who answered the questions carefully were included in the analyses, respondents with a fit statistic (root likelihood) below .247 were omitted from the analyses as a fit statistic below this score would suggest random answers to the choice tasks.^28^ In addition to the RISs, most‐minus‐least counts were calculated to confirm the rankings of the attributes. To adjust for an imbalance in the number of times an attribute was shown to each respondent in the best‐worst scaling, the most‐minus‐least counts were divided by the frequency of each attribute being included in the questionnaire across the four versions.^24^

Multivariable linear regression analyses were conducted with SPSS for Windows version 24 to assess whether the RISs of the 27 attributes differed between neurologists and MS nurses while controlling for sex and three professional characteristics. Two characteristics indicated the amount of experience in treating patients with MS: the number of years treating patients with MS dichotomized into ≤15 years or >15 years and the number of patients with MS treated yearly dichotomized into ≤150 patients per year or >150 patients treated per year. The third characteristic denoted the extent to which the healthcare professional was specialized in treating patients with MS on a continuous scale and was defined as the number of patients with MS treated as proportion of the total number of patients treated.

To assess whether the RISs assigned by the neurologists and nurses to the 27 attributes differed from the RISs assigned by the patients with MS, hierarchical Bayes analyses were conducted including responses of the neurologists and nurses, and responses of patients obtained in a previous study.^8^ A second linear regression model was built to control for age, sex and level of education in the function between the respondent type (i.e. healthcare professional or patient) and the RISs of the 27 attributes. Level of education was dichotomized into respondents who had completed primary and/or secondary school only and respondents who had completed any additional type of tertiary education. The patients’ perspective was obtained from a previous study among 185 patients with relapsing‐remitting MS or clinically isolated syndrome, and with a range in experience with DMDs (from having never used a DMD to having used more than three different DMDs) and duration of diagnosis. Further detailed description of the patient recruitment and the patients characteristics can be found elsewhere.^8^

## 3 RESULTS

Of the 120 neurologists and an estimated 140 nurses contacted, 79 healthcare professionals accessed the questionnaire between 20 November 2015 and 8 February 2016. In total, 62 healthcare professionals completed the questionnaire (estimated overall completion rate of 24%), of which two healthcare professionals did not meet the inclusion criteria because they were working in Belgium (n=1) or did not treat patients with MS on a regular basis (n=1). The sample of 60 healthcare professionals that met the inclusion criteria consisted of 27 (45%) neurologists and 33 (55%) nurses. Table 1 presents their characteristics. Twenty‐four (89%) neurologists reported to be specialized in MS. Of the MS nurses, 23 (70%) MS‐specialized nurses, one (3%) nurse consultant MS, seven (21%) nurse practitioners MS and two (6%) physician assistants MS completed the questionnaire. The median proportion of patients with MS expressed as a percentage of the total number of patients treated was 20% among neurologists and 50% among nurses, of which eight nurses reported to only treat patients with MS.

**Table 1:** Characteristics of the health care professionals

	All healthcare professionals (N=60)	Neurologists (N=27)	Nurses and physician assistants (N=33)
Sex, N (%)			
Male	21 (35.0)	20 (74.1)	1 (3.0)
Female	39 (65.0)	7 (25.9)	32 (97.0)
Age (in years)			
mean (SD)	47.6 (8.7)	49.7 (9.2)	45.9 (8.1)
Range	30‐64	35‐64	30‐59
Work function, N (%)			
MS‐specialized neurologist	24 (40)	24 (88.9)	‐
General neurologist	3 (5.0)	3 (11.1)	‐
MS nurse	23 (38.3)	‐	23 (69.7)
Nurse practitioner MS	7 (11.7)	‐	7 (21.2)
Physician assistant	2 (3.3)	‐	2 (6.1)
Nurse consultant	1 (1.7)	‐	1 (3.0)
Work experience in years, mean (SD)			
≤15	45 (75.0)	16 (59.3)	29 (87.9)
>15	15 (25.0)	11(40.7)	4 (12.1)
Number of MS patients treated yearly, mean (SD)			
≤150	24 (40.0)	11 (40.7)	13 (39.4)
>150	36 (60.0)	16 (59.3)	20 (60.6)
Proportion MS patients from total number of patients treated (in %)			
Median (IQR)	40 (20‐67.5)	20 (10‐40)	50 (40‐95)

IQR, interquartile range; MS, multiple sclerosis; SD, standard deviation.

As all fit statistics of the questionnaires were above .247 (mean .557; range .341, .769), indicating that none of the healthcare professionals answered the choice tasks completely at random, all completed questionnaires were included in the analyses. The ranking of the DMD attributes according to the mean RISs showed that healthcare professionals regarded safety, that is risks of life‐threatening or severely disabling adverse events, as most important (mean RIS [SD]: 9.29 [0.92]), but was followed closely by effect on disability progression, effect on quality of life and effect on relapse rate with only small differences between the RISs (mean RIS [SD]: 9.27 [1.58], 9.19 [0.83] and 8.89 [0.88], respectively). Other highly ranked attributes were effect on development of plaques in the brain (i.e. MS activity on MRI), severity of side effects and the effect on severity of relapses. Ten DMD attributes were of little or no importance in the decision for the healthcare professionals with mean RIS below 1.0, including the required monitoring and administration frequency. The results of the adjusted most‐minus‐least counts (Table S1 Most‐minus‐least counts) did not substantially affect the rankings obtained based on the hierarchical Bayes analyses and therefore confirmed the results. Of the 60 neurologists and nurses, two (3%) neurologists reported additional DMD attributes that were not included in the best‐worst scaling: teratogenic properties of the DMD and the certainty of achieving the effects of the DMD.

### 3.1 Comparison of neurologists with MS nurses

Only few differences were found between neurologists and nurses (Table 2). When comparing neurologists to nurses, relatively large differences in mean RISs (absolute difference in RISs of 1 or more) were found in effect on current MS symptoms, effect on life expectancy and total costs of the DMD, which were higher for the neurologists, and interaction with other medication, which was higher for nurses. However, when controlling for sex, the years of work experience, the number of patients with MS treated each year and the degree of focus on MS treatment, only a significant difference was found between neurologists and nurses in the low ranked attributes total cost of the DMD (b=−1.27, *P*=.015) and interaction with other medication (b=2.09, *P*=.007). These differences resulted in quite substantial shifts in ranking: total cost of the DMD was ranked 17th by neurologists (RIS=1.22) and 25th by nurses (RIS=0.16), and interaction with other medication was ranked 18th by neurologists (RIS=1.10) and 13th by nurses (RIS=2.97). Based on their RISs though, these attributes did not influence the treatment decision severely.

**Table 2:** Relative importance scores of the DMD attributes in the treatment decision according to neurologists and nurses, and results of the multivariable analyses for work function (neurologist or nurse) while controlling for other variables

Attribute	Neurologists		Nurses		Difference in RIS according to profession		
	Rank	Relative importance score Mean (SD)	Rank	Relative importance score Mean (SD)	b [95% CI]	t(59)	p
Effect on disability progression	1	9.41 (1.78)	3	9.17 (1.41)	−1.14 [−2.43. 0.16]	−1.76	.084
Safety	2	9.19 (0.83)	1	9.38 (0.98)	−0.03 [−0.77. 0.72]	−0.07	.943
Effect on quality of life	3	9.02 (0.90)	2	9.34 (0.74)	0.58 [−0.09. 1.26]	1.73	.089
Effect on relapse rate	4	8.98 (0.84)	4	8.81 (0.92)	−0.03 [−0.77. 0.72]	−1.61	.113
Effect on development of plaques in the brain	5	8.16 (1.23)	5	8.55 (1.66)	0.12 [−1.11. 1.34]	0.19	.851
Severity of side effects	6	8.14 (1.20)	6	8.00 (0.92)	−0.48 [−1.37. 0.40]	−1.10	.276
Effect on the severity of relapse	7	7.08 (2.11)	7	7.52 (1.56)	0.41 [−1.10. 1.91]	0.54	.590
Effect on current MS symptoms	8	6.45 (2.83)	9	5.13 (2.97)	−0.40 [−2.74. 1.93]	−0.35	.731
Effect on life expectancy	9	5.71 (3.19)	12	4.23 (3.07)	−0.78 [−3.40. 1.84]	−0.60	.554
Uncertainty about long‐term consequences	10	5.63 (1.98)	11	4.72 (2.20)	−1.34 [−3.09. 0.41]	−1.53	.131
Influence on lifestyle	11	4.94 (0.61)	8	5.14 (0.59)	0.21 [−0.28. 0.71]	0.86	.392
Type of side effects	12	4.65 (1.42)	10	4.79 (1.56)	−0.09 [−1.27. 1.10]	−0.14	.886
Duration of side effects	13	2.42 (1.07)	14	2.83 (1.72)	0.59 [−0.64. 1.82]	0.97	.338
Pace of effect	14	2.02 (1.90)	15	2.83 (2.15)	0.74 [−0.92. 2.41]	0.90	.374
Mode of administration	15	1.50 (1.60)	16	1.26 (1.22)	−0.73 [−1.90. 0.43]	−1.26	.213
Insurance coverage	16	1.37 (2.60)	18	1.04 (1.97)	−0.28 [−2.17. 1.62]	−0.29	.771
Total DMD costs	17	1.22 (1.80)	25	0.16 (0.26)	−1.27 [−2.29. −0.25]^a^	−2.50	.015
Interaction with other medication	18	1.10 (0.83)	13	2.97 (2.32)	2.09 [0.60. 3.59]^a^	2.81	.007
Required monitoring	19	0.78 (1.01)	19	0.92 (1.59)	0.79 [−0.24. 1.81]	1.54	.129
Mode of action of DMD	20	0.67 (1.44)	17	1.17 (1.82)	0.75 [−0.55. 2.05]	1.15	.254
Frequency of administration	21	0.66 (0.62)	20	0.85 (0.70)	0.19 [−0.36. 0.74]	0.70	.488
Duration of administration	22	0.35 (0.35)	24	0.17 (0.14)	−0.05 [−0.25. 0.14]	−0.55	.586
Further development of DMD	23	0.25 (0.64)	21	0.43 (0.65)	0.26 [−0.26. 0.78]	1.01	.316
Use of DMD among other MS patients	24	0.11 (0.10)	23	0.20 (0.17)	0.10 [−0.02. 0.21]	1.61	.114
Ease of travelling	25	0.10 (0.32)	26	0.09 (0.09)	0.05 [−0.14. 0.23]	0.53	.598
Composition of DMD	26	0.08 (0.14)	22	0.29 (0.51)	0.29 [−0.04. 0.61]	1.77	.082
Contact person at pharmaceutical company	27	0.01 (0.03)	27	0.02 (0.04)	−0.01 [−0.03. 0.02]	−0.48	.634

CI, confidence interval; DMD, disease‐modifying drug; MS, multiple sclerosis; RIS, relative importance score; SD, standard deviation.

a *P*<.05.

### 3.2 Comparison of healthcare professionals (neurologists and MS nurses) with patients

**Figure 2:**
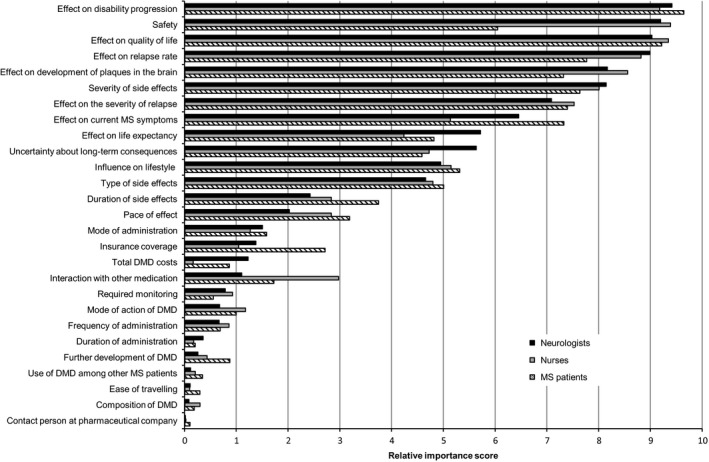
Relative importance scores of attributes of disease‐modifying drugs according to neurologists, nurses and patients with multiple sclerosis

When controlling for age, sex and level of education, there were significant differences found between the RISs of the healthcare professionals and the patients with MS for six attributes at an alpha of .05: effect on relapse rate and safety were more important for neurologists and nurses, while effect on current MS symptoms, pace of effect, insurance coverage and further development of the DMD were more important for the patients in the decision about DMDs. Furthermore, while the difference in RIS of 1.01 between the healthcare professionals and patients for influence on lifestyle was quite substantial, this difference was not significant when controlling for age, sex and level of education (*P*=.063). For safety, the difference was the most notable: the RIS for patients was substantially lower than for neurologists and nurses (b=−2.59, *P*<.001), making safety the fourth most important attribute for neurologists and nurses, and only the eighth most important attribute for patients. Table 3 presents the results of the multivariable regression analyses for all 27 attributes. Figure 2 presents the relative importance score of each attribute according to patients with MS and neurologists and MS nurses.

**Table 3:** Relative importance scores of the DMD attributes in the treatment decision according to respondent type (i.e. neurologists/nurses or MS patients), and results of the multivariable analyses for respondent type while controlling for other variables

Attribute	Neurologists and nurses		MS patients		Difference in RIS according to respondent type (neurologists and nurses vs MS patients)		
	Rank	Relative importance score mean (SD)	Rank	Relative importance score mean (SD)	b [95% CI]	t(244)	*P*
Effect on disability progression	1	9.50 (1.31)	1	9.64 (1.16)	0.19 [−0.20, 0.57]	0.96	.336
Effect on quality of life	2	9.23 (0.97)	2	9.21 (1.45)	0.07 [−0.36, 0.50]	0.31	.755
Effect on relapse rate	3	8.71 (1.31)	3	7.76 (2.58)	−0.88 [−1.62, −0.13]^a^	−2.32	.021
Safety	4	8.69 (1.80)	8	6.04 (2.95)	−2.59 [−3.45, −1.72]^a^	−5.90	<.001
Effect on development of plaques in the brain	5	8.00 (1.83)	7	7.31 (2.52)	−0.49 [−1.24, 0.27]	−1.27	.204
Severity of side effects	6	7.87 (1.09)	4	7.63 (2.11)	−0.32 [−0.93, 0.29]	−1.02	.307
Effect on the severity of relapse	7	7.51 (1.75)	5	7.39 (2.32)	−0.08 [−0.77, 0.61]	−0.23	.822
Effect on current MS symptoms	8	6.28 (2.52)	6	7.32 (1.97)	1.13 [0.46, 1.81]^a^	3.31	.001
Uncertainty about long‐term consequences	9	5.45 (2.31)	12	4.58 (2.76)	−0.82 [−1.66, 0.03]	−1.91	.057
Effect on life expectancy	10	4.97 (3.07)	11	4.81 (3.13)	0.11 [−0.87, 1.10]	0.23	.818
Type of side effects	11	4.70 (1.81)	10	5.00 (2.71)	0.07 [−0.73, 0.87]	0.17	.863
Influence on lifestyle	12	4.30 (2.68)	9	5.31 (2.92)	0.86 [−0.05, 1.77]	1.87	.063
Duration of side effects	13	3.43 (1.44)	13	3.74 (1.97)	0.19 [−0.40, 0.79]	0.64	.521
Pace of effect	14	2.51 (2.00)	14	3.18 (2.19)	0.73 [0.04, 1.42]^a^	2.09	.038
Interaction with other medication	15	1.88 (1.76)	16	1.72 (1.86)	0.00 [−0.57, 0.56]	−0.01	.992
Insurance coverage	16	1.47 (2.34)	15	2.71 (2.87)	1.05 [0.17, 1.93]^a^	2.34	.020
Mode of administration	17	1.37 (1.78)	17	1.58 (2.69)	0.00 [−0.80, 0.79]	0.00	.997
Mode of action of DMD	18	0.87 (1.44)	18	0.99 (1.25)	0.17 [−0.24, 0.57]	0.80	.422
Required monitoring	19	0.77 (1.27)	22	0.55 (1.18)	−0.22 [−0.60, 0.17]	−1.12	.264
Frequency of administration	20	0.71 (0.67)	21	0.68 (1.38)	−0.13 [−0.53, 0.27]	−0.65	.518
Total DMD costs	21	0.54 (1.05)	20	0.86 (1.27)	0.24 [−0.15, 0.63]	1.21	.226
Further development of DMD	22	0.44 (0.69)	19	0.87 (0.94)	0.49 [0.22, 0.77]^a^	3.49	.001
Duration of administration	23	0.23 (0.21)	25	0.20 (0.24)	−0.02 [−0.09, 0.05]	−0.50	.615
Composition of DMD	24	0.20 (0.41)	26	0.18 (0.36)	−0.04 [−0.16, 0.08]	−0.59	.553
Use of DMD among other MS patients	25	0.19 (0.21)	23	0.34 (0.56)	0.14 [−0.02, 0.29]	1.68	.095
Ease of travelling	26	0.14 (0.45)	24	0.29 (0.87)	0.08 [−0.18, 0.33]	0.60	.552
Contact person at pharmaceutical company	27	0.03 (0.05)	27	0.10 (0.28)	0.06 [−0.02, 0.14]	1.47	.142

CI, confidence interval; DMD, disease‐modifying drug; MS, multiple sclerosis; RIS, relative importance score; SD, standard deviation.

a *P*<.05.

## 4 DISCUSSION

In this study, a best‐worst scaling was used to assess the neurologists’ and MS nurses’ perspectives on the importance of 27 DMD attributes in the decision about DMD treatments, which enabled comparison of the healthcare professionals’ perspective to the patients’ perspective. A best‐worst scaling is a rigorous method for assessing preferences often used in healthcare research,^29^ but, to our knowledge, has not been used before to prioritize the information about treatment options for the development of a decision aid.

This study found that neurologists and MS nurses ranked safety, effect on disability progression, effect on quality of life and effect on relapse rate highest. These attributes were thus considered as most influential in the decision making. Next in ranking were attributes focused on other effects and on side effects. Influence on lifestyle was the highest ranked (10th) attribute not directly related to benefits and possible harms but was only awarded a mean RIS of almost half of the most important attribute. One other study has evaluated the neurologists’ perspective on important attributes in decisions about DMD by asking neurologists in the United States to rank five attributes. This study found, similarly to the current study, that efficacy and safety were the most important attributes, followed by tolerability, patient preferences and, lastly, convenience.^22^ Within the group of healthcare professionals, we expected some differences in importance of attributes between neurologists and nurses as a result of a difference in the relationship with the patient. However, in our study we found only statistical differences in two low ranked attributes, suggesting that neurologists and MS nurses do not differ much in their perspective about what to consider in the decision about DMDs.

The healthcare professionals and the patients both valued effect on disease progression, quality of life and relapse rate highly, which is not unexpected because these are the primary aims of DMD treatment. The most notable difference between the two perspectives was found in the importance of safety. The healthcare professionals gave significantly more value to this attribute compared with patients. More specifically, safety was ranked as fourth most important by healthcare professionals, while ranked only eighth by patients, who gave more value to other attributes focused on effectiveness and the severity of more common side effects. In the process of shared decision making, this could be an issue. Healthcare professionals may, for example, be less inclined than patients to choose a DMD for which experience and research has shown risks for life‐threatening or severely disabling side effects, such as progressive multifocal leukoencephalopathy. Motivations of patients, neurologists and nurses for the importance of the attributes were not elicited in the questionnaire; therefore, we can only speculate about the reasons for the difference found in the importance of the attributes, in particularly safety. A first explanation for the difference in the importance of safety might be that healthcare professionals have a different understanding of the seriousness of the risks and consequences than patients. Second, patients might be willing to take more risk, as a previous study showed that patients were willing to accept larger risks than the risks associated with the DMDs available at that time, in exchange for effectiveness in reducing relapse rate and disability progression.^9^ Healthcare professionals may be less inclined to take these risks, for example, because of forensic and legal liability in case of incidents of serious adverse events.

This study shows which attributes are most important in the decision, and that the rankings of the attributes are overall quite similar for neurologists, MS nurses and MS patients, with the exception of a few attributes—especially safety. It should be noted that the rankings reflect the average preferences. Therefore, the discrepancies do not necessarily play a role in every consultation. However, incorporating the patient's preferences in the decision is an essential part of reaching a shared decision, which may be complicated if the healthcare professional and patient do not agree on the importance of some attributes in the decision. Patient decision aids can, besides provide information, also support the patient and the healthcare professional in eliciting and discussing the patient's preferences.^30^ The results of this study contribute to the development of an MS decision aid by indicating the attributes—those attributes that both patients and healthcare professionals find most important and those attributes that are substantially more important for either patients or healthcare professionals—about which information and preference elicitation questions should be included in the decision aid to enable effective support for reaching a shared decision. Moreover, this study shows that a best‐worst scaling could be a useful tool in the future development of other interventions for supporting shared decision making, such as patient decision aids. In particular when there is a risk of developing overly cognitive burdensome tools, a best‐worst scaling may be useful in selecting the most essential attributes.

Some limitations of this study need to be considered. Only an estimated 24% of the contacted healthcare professionals completed the questionnaire. Possibly, more healthcare professionals with a more positive attitude towards research responded because of our recruitment methods. Therefore, the sample might not perfectly represent all MS neurologists and MS nurses in the Netherlands because they may have been better informed about the latest DMD developments, and they might have had different preferences for DMD attributes accordingly. Another limitation could be the small sample size, but the number of eligible healthcare professionals for this study is also limited. The Netherlands counts about 80 hospitals of which most hospitals have one, two or three neurologists with a focus on MS and one or two MS nurses, and we made efforts to invite all of them to participate. The sample size limited the ability to conduct a latent class analysis. In future research, evaluation of heterogeneity in preferences using latent class analysis could potentially provide more insight into groups of healthcare professionals with similar preferences. Furthermore, to compare the perspectives of the neurologists and the MS nurses with those of the patients, the best‐worst scaling for both parties included the same attributes. These attributes were, however, identified through focus groups among patients only, and may not be the same attributes that the neurologists and MS nurses would have reported if focus groups among them would have been performed. Nevertheless, only two neurologists reported other attributes that were not included in the best‐worst scaling—teratogenic properties and uncertainty whether effects would be achieved—to be of importance as well. Another limitation is that it was necessary to ask different questions in the best‐worst scaling for healthcare professionals compared with the patients. Both groups were asked to choose the most and least important attribute for decision making, but patients were expected to answer this question according to their individual situation, while healthcare professionals were asked to answer the question for patients with MS in general. This difference in framing of the question may have led to differences in what is regarded as important in the decision. Additionally, because of small deviations from normality of the residuals and homoscedasticity found in the regression analyses, generalization of the found differences in total costs of the DMD and interaction with other medication between neurologists and nurses and the differences in effect on relapse rate, insurance coverage, and further development of the DMD between the healthcare professionals and the patients beyond our sample should be done with caution. Lastly, it is uncertain whether our results may be transferred to other settings as the healthcare systems in other settings may differ. Therefore, some attributes, such as DMD costs, may be more important for the decision in other settings. Other limitations that apply to the best‐worst scaling method and the results of the patients’ perspective have been reported elsewhere.^8^

## 5 CONCLUSION

This study showed that safety, effect on disability progression and effect on quality of life were the most influential attributes of DMDs for healthcare professionals in the treatment decision. The importance of these and other highly ranked attributes did not differ between neurologists and nurses when making a decision for patients with MS in general. Additionally, the average healthcare professionals’ perspective and the average patients’ perspective agree that the ability of DMDs to reduce disability progression and maintain or improve quality of life are the most important attributes of a DMD. The perspectives differ however considerably about the importance of safety. These results provide valuable information for the development of interventions to support shared decision making. This study also demonstrates the feasibility of combining focus groups and a best‐worst scaling to identify important attributes that could later be included in a patient decision aid. A best‐worst scaling could be an interesting method when having to restrict the number of attributes for inclusion in a patient decision aid.

## CONFLICT OF INTEREST

During the time of the study, PJ Jongen has received honoraria from Bayer, Merck and Teva for contributions to symposia as a speaker or for consultancy activities. The other authors have no other conflicting interest to declare.

## Supporting information


** **
