# Supplementary material for: Comparison of preferences of healthcare professionals and MS patients for attributes of disease‐modifying drugs: A best‐worst scaling
Source: Health Expect. 2017 Jul 21;21(1):171–80. doi: 10.1111/hex.12599 (PMC5750752; doi:10.1111/hex.12599)
Supplement: Supplementary file 1 [file HEX-21-171-s001.docx]

Supporting information

**Table**. Results of the most-minus-least (M-L) counts. M-L counts according to healthcare professionals combined, neurologists, nurses and MS patients are presented per attribute. Attributes are ranked according to healthcare professionals’ score for M-L as proportion of the frequency of the attribute presented in the questionnaire.

|  | Healthcare professionals (N=60) | | | | Neurologists (N=27) | | | | MS nurses (N=33) | | | | MS patients (N=185) | | | |
| --- | --- | --- | --- | --- | --- | --- | --- | --- | --- | --- | --- | --- | --- | --- | --- | --- |
| Attribute | M count | L count | M-L count | % | M count | L count | M-L count | % | M count | L count | M-L count | % | M count | L count | M-L count | % |
|  |  |  |  | M-L^1^ |  |  |  | M-L^1^ |  |  |  | M-L^1^ |  |  |  | M-L^1^ |
| Effect on disability progression | 147 | 2 | 145 | 74.0% | 81 | 1 | 80 | 87.9% | 91 | 1 | 90 | 85.7% | 394 | 2 | 392 | 65.9% |
| Safety | 107 | 0 | 107 | 59.4% | 46 | 0 | 46 | 56.8% | 58 | 0 | 58 | 58.6% | 177 | 30 | 147 | 24.6% |
| Effect on quality of life | 115 | 0 | 115 | 58.4% | 45 | 0 | 45 | 51.7% | 59 | 0 | 59 | 53.6% | 369 | 2 | 367 | 61.1% |
| Effect on relapse rate | 103 | 2 | 101 | 53.4% | 49 | 1 | 48 | 57.1% | 58 | 1 | 57 | 54.3% | 273 | 18 | 255 | 42.4% |
| Effect on development of plaques in the brain | 82 | 5 | 77 | 42.8% | 29 | 2 | 27 | 33.3% | 41 | 2 | 39 | 39.4% | 203 | 22 | 181 | 32.6% |
| Severity of side effects | 54 | 0 | 54 | 30.0% | 25 | 0 | 25 | 30.9% | 31 | 0 | 31 | 31.3% | 207 | 2 | 205 | 36.9% |
| Effect on severity of relapses | 56 | 2 | 54 | 28.6% | 21 | 1 | 20 | 23.8% | 28 | 1 | 27 | 25.7% | 208 | 11 | 197 | 32.2% |
| Effect on current MS symptoms | 61 | 21 | 40 | 22.2% | 32 | 5 | 27 | 33.3% | 35 | 8 | 27 | 27.3% | 207 | 27 | 180 | 30.3% |
| Type of side effects | 36 | 3 | 33 | 18.3% | 19 | 2 | 17 | 21.0% | 25 | 2 | 23 | 23.2% | 118 | 17 | 101 | 18.2% |
| Effect on life expectancy | 55 | 24 | 31 | 15.8% | 33 | 8 | 25 | 27.5% | 37 | 14 | 23 | 21.9% | 149 | 70 | 79 | 14.2% |
| Uncertainty about long-term consequences | 33 | 8 | 25 | 13.9% | 19 | 3 | 16 | 19.8% | 21 | 3 | 18 | 18.2% | 109 | 56 | 53 | 9.5% |
| Influence on life style | 40 | 17 | 23 | 12.2% | 14 | 11 | 3 | 3.6% | 16 | 12 | 4 | 3.8% | 190 | 75 | 115 | 18.8% |
| Pace of effect | 26 | 21 | 5 | 2.6% | 11 | 12 | -1 | -1.1% | 14 | 14 | 0 | 1.0% | 80 | 46 | 34 | 9.1% |
| Duration of side effects | 14 | 9 | 5 | 2.5% | 3 | 4 | -1 | -1.1% | 6 | 5 | 1 | 0.0% | 76 | 22 | 54 | 6.1% |
| Interaction with other medication | 13 | 16 | -3 | -1.5% | 1 | 11 | -10 | -11.5% | 2 | 12 | -10 | -9.1% | 37 | 75 | -38 | -6.8% |
| Mode of administration | 10 | 32 | -22 | -11.1% | 5 | 13 | -8 | -9.0% | 5 | 16 | -11 | -10.1% | 65 | 177 | -112 | -18.8% |
| Frequency of administration | 13 | 41 | -28 | -15.6% | 2 | 18 | -16 | -19.8% | 3 | 19 | -16 | -16.2% | 38 | 207 | -169 | -28.1% |
| Required monitoring | 7 | 49 | -42 | -23.3% | 1 | 21 | -20 | -24.7% | 4 | 25 | -21 | -21.2% | 18 | 242 | -224 | -36.6% |
| Mode of action of DMD | 22 | 70 | -48 | -23.8% | 9 | 39 | -30 | -17.9% | 11 | 43 | -32 | -21.9% | 56 | 173 | -117 | -2.2% |
| Insurance coverage | 11 | 56 | -45 | -24.4% | 5 | 20 | -15 | -34.5% | 6 | 29 | -23 | -29.1% | 89 | 102 | -13 | -21.1% |
| Total costs | 6 | 72 | -66 | -33.3% | 6 | 17 | -11 | -12.4% | 6 | 29 | -23 | -21.1% | 16 | 149 | -133 | -24.0% |
| Use of DMD among other MS patients | 0 | 67 | -67 | -37.2% | 0 | 31 | -31 | -38.3% | 0 | 38 | -38 | -38.4% | 7 | 215 | -208 | -34.8% |
| Duration of administration | 1 | 78 | -77 | -38.9% | 0 | 25 | -25 | -28.1% | 0 | 37 | -37 | -33.9% | 10 | 277 | -267 | -44.9% |
| Further development of DMD | 2 | 85 | -83 | -42.1% | 1 | 45 | -44 | -50.6% | 1 | 55 | -54 | -49.1% | 24 | 159 | -135 | -24.3% |
| Composition of DMD | 4 | 102 | -98 | -50.0% | 1 | 53 | -52 | -57.1% | 2 | 57 | -55 | -52.4% | 9 | 310 | -301 | -54.2% |
| Ease of traveling | 1 | 105 | -104 | -57.8% | 1 | 50 | -49 | -60.5% | 1 | 57 | -56 | -56.6% | 13 | 322 | -309 | -50.5% |
| Contact person at pharmaceutical company | 1 | 133 | -132 | -73.3% | 0 | 66 | -66 | -81.5% | 0 | 81 | -81 | -81.8% | 3 | 337 | -334 | -55.6% |

^1^ M-L as proportion of frequency of which attribute was presented to the respondents. If positive, the respondents chose this attribute more often as most important attribute than as least important attribute. If negative, the respondents chose the attribute more often as least important than as most important.

Note. DMD = disease-modifying drug, MS = multiple sclerosis, M counts = frequency of attribute chosen as most important attribute, L counts = frequency of attribute chosen as least important attribute, M-L count = most-minus-least counts
